# The expected labor progression after labor augmentation with oxytocin: A retrospective cohort study

**DOI:** 10.1371/journal.pone.0205735

**Published:** 2018-10-31

**Authors:** Lin Zhang, James Troendle, D. Ware Branch, Matthew Hoffman, Jun Yu, Lixia Zhou, Tao Duan, Jun Zhang

**Affiliations:** 1 Gynecology and Obstetrics Department, Xinhua Hospital, Shanghai Jiao Tong University School of Medicine, Shanghai, China; 2 MOE-Shanghai Key Lab of Children’s Environmental Health, Xinhua Hospital, Shanghai Jiao Tong University School of Medicine, Shanghai, China; 3 National Heart Lung and Blood Institute, National Institutes of Health, Bethesda, Maryland, United States of America; 4 Intermountain Healthcare and University of Utah Health Sciences, Salt Lake, Utah, United States of America; 5 Christiana Care Health System, Newark, Delaware, United States of America; 6 Shanghai First Maternity and Infant Hospital, Shanghai, China; Indiana University School of Medicine, UNITED STATES

## Abstract

**Objective:**

To describe labor progression patterns with oxytocin for augmentation in women who achieve vaginal delivery; and to determine how long one should wait with effective uterine contraction before labor arrest can be diagnosed.

**Design:**

Population-based retrospective cohort study.

**Population:**

The final sample involved 8,988 women with singleton gestation, term live birth, vertex presentation, no previous cesarean section, vaginal delivery, and neonatal Apgar score at 5 minutes at 7 or higher, and complete information on oxytocin augmentation in 2005–2007.

**Methods:**

Linear interpolation was used from the vaginal exam records for each woman to estimate the cervical dilation when oxytocin was started and the highest dose was first reached by parity. We used survival methods to estimate quartiles of the traverse time distributions of cervical dilation.

**Main outcome measures:**

Duration of labor under oxytocin augmentation.

**Results:**

When oxytocin was just started, it took a long time to observe cervical dilation. The 50^th^(95^th^) centiles of the time interval from 4 to 5 cm, 5 to 6 cm, and 6 to 10 cm dilation were 2.9(8.8) hr, 1.7(5.8) hr, and 2.1(6.0) hr in nulliparas; and 3.1(10.1) hr, 1.9(8.0) hr, and 1.7(6.2) hr in multiparas. After effective uterine contractions were achieved under oxytocin, labor progressed much faster. The corresponding values were 0.7(2.4)hr, 0.5(1.5)hr, and 0.5(1.5)hr in nulliparas; and 0.6(1.9)hr, 0.4(1.1)hr, and 0.4(0.9)hr in multiparas. Low- and high-dose oxytocin regimens had similar effects on labor.

**Conclusion:**

When oxytocin is just started for labor augmentation in early first stage, it may take up to 10 hours for the cervix to dilate by 1 cm. Once effective uterine contractions are achieved and the cervix is dilated more than 5 cm, cervical dilation to the next centimeter occurs within 2 hrs in both nulliparas and multiparas in 95% of the cases. High- and low-dose oxytocin had a similar impact on labor progression in augmented labor.

## Introduction

Labor arrest remains the dominant indication for primary caesarean sections in most countries, accounting for one-third of all caesarean deliveries in the U.S.[1] Oxytocin is frequently employed to address dystocia.[2] However, our basic knowledge on how labor progresses when it is augmented by oxytocin is still very limited. For example, once oxytocin administration is initiated, how long it may take before one expects to see perceivable cervical change is poorly defined. More importantly, after effective uterine contraction is achieved, how long one should wait before labor arrest can be diagnosed needs further clinical evidence. Unfortunately, the wide variation in oxytocin dose regimen complicates an already challenging clinical issue in labor management. To our best knowledge, no study with a sufficiently large sample size has described in-depth the labor progression after oxytocin augmentation.

The recent American Congress of Obstetricians and Gynecologists(ACOG) guidelines on dystocia management recommend that a minimum of 4 hours of effective uterine contractions with oxytocin augmentation should be allowed before labor arrest is declared and cesarean delivery performed.[3] NICE guideline advises the woman to have a vaginal examination 4 hours after starting oxytocin in established labour, if cervical dilatation has increased by less than 2 cm after 4 hours of oxytocin, further obstetric review is required to assess the need for caesarean section.[4] As labor progression accelerates in the active phase, does the 4-hour rule apply to the early as well as late active phase? The purpose of this study was to use contemporary labor data in a large number of parturients receiving oxytocin to describe labor progression patterns with oxytocin for augmentation.

## Materials and methods

The Consortium on Safe Labor was a retrospective observational study from 2002 to 2008. A detailed description was provided elsewhere.[5] The study collected comprehensive information on contemporary labor and delivery practices in multiple institutions across the US. Participating institutions extracted detailed information from their electronic medical records on maternal demographic characteristics, medical history, reproductive and prenatal history, labor and delivery summary, postpartum and newborn information. Information from the neonatal intensive care unit was linked to the newborn records. Data on oxytocin use were extracted from a medication database. The Institutional Review Boards of all participating institutions approved this project.

**Fig 1:**
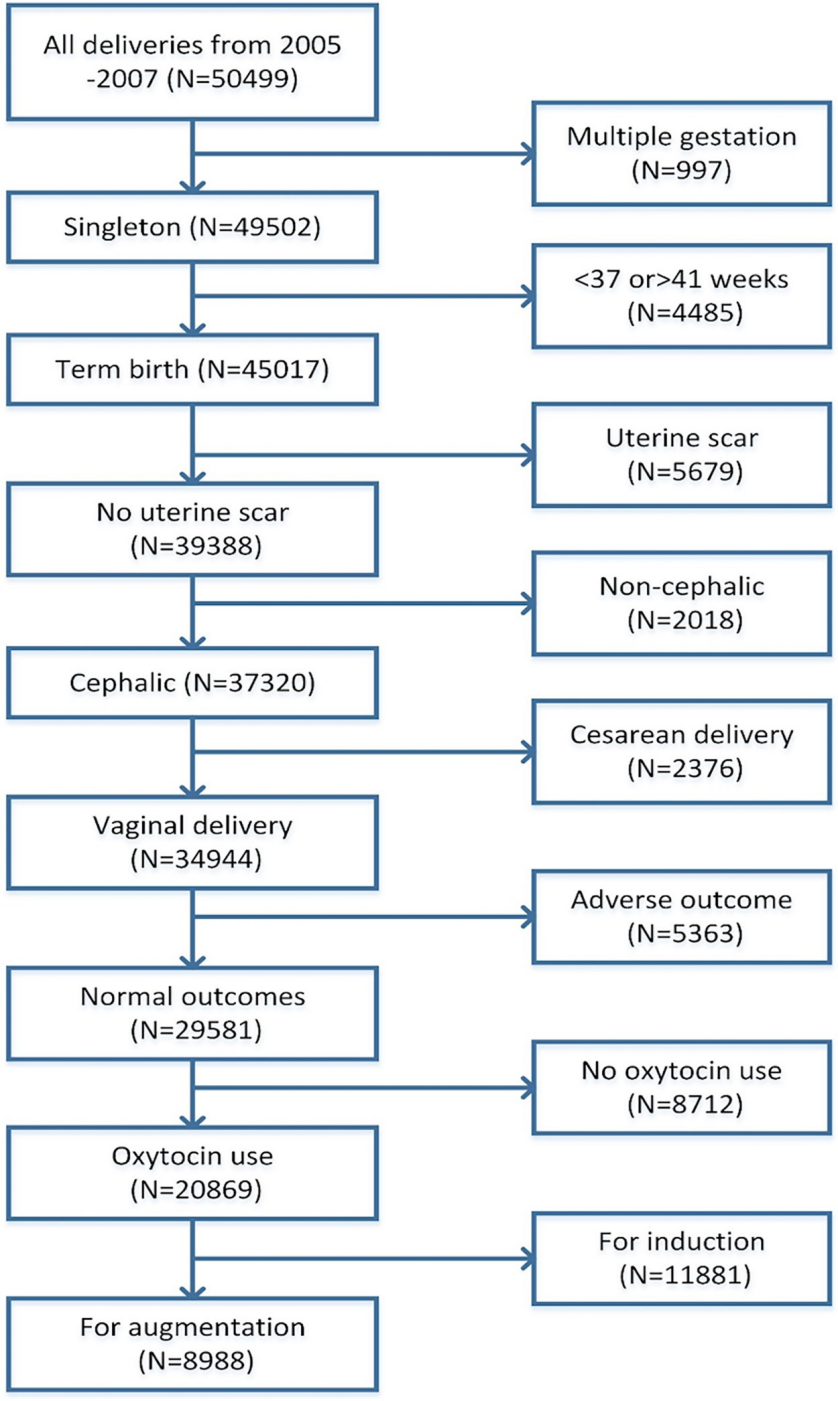
Selection of study population.

A total of 228,562 births were collected in the database. Not all participating hospitals provided detailed information on oxytocin regimen, dose, and increments. We chose 5 institutions from Intermountain Healthcare that had such data (N = 50,499). The following subjects were further selected: singleton gestation, term live birth, no previous cesarean section, vertex presentation (excluding face and brow), vaginal delivery, normal perinatal outcome, and use of oxytocin for labor augmentation (Fig 1). To address the fetal safety concern, we restricted to women who achieve vaginal delivery and normal perinatal outcomes by excluding newborns with congenital anomalies, neonatal intensive care unit admission or birth trauma, neonatal Apgar score at 5 minutes less than 7, and maternal severe complications such as postpartum hemorrhage or 3rd/4th degree laceration, leaving 8,988 women for analysis.

Detailed oxytocin regimens in these hospitals have been described previously.[6] Briefly, both high- and low-dose oxytocin regimens were used. A high-dose regimen was defined as a starting dose at 4 mU/min and increments of 4 mU/min within every 30 min. Starting dosages at 1–2 mU/min and increments of 1–2 mU/min within 30 min were defined as low-dose regimen. It should be noted we did not have specific information on whether and when the uterus had achieved effective contraction with oxytocin; nor did we have a uniform definition of “effective uterine contraction”. Instead, we used the time when the oxytocin reached the highest dose for the first time as a surrogate indicator for feasible effective uterine contraction. This assumption may be reasonable as all our subjects achieved vaginal delivery in this analysis.

### Statistical analysis

To analyze duration of labor we first used linear interpolation from the vaginal exam records for each woman to estimate the cervical dilation when oxytocin was started. Next, these estimates were rounded to the nearest integer centimeter. We then considered separately each integer centimeter increment in cervical dilation (4–5 cm, 5–6 cm, etc.), restricting to labors where the woman was estimated to start oxytocin prior to the end of the interval. Since the exact times to traverse each interval of cervical dilation is unknown for each woman due to interval censoring, we used survival methods described previously[7] to estimate quartiles of the traverse time distributions. We ran models separated by parity (nulliparas vs multiparas) allowing us to obtain percentile estimates for subsets based on those factors. The bootstrap (100 with-replacement samples of size equal to the observed number of labors) was used to estimate standard deviations of our estimated percentiles (not shown).

To analyze the dilation when the highest dose of oxytocin was first reached during labor, we started by identifying the first time the highest dose of oxytocin was recorded for each labor. Linear interpolation was then used to estimate from the vaginal exam records for each woman the cervical dilation when the highest dose was first reached. We then used the rounded dilation at oxytocin start to separately analyze those labors where oxytocin started at a given rounded cm dilation (2, 3, etc.).

The Consortium on Safe Labor was conducted by the National Institutes of Health (NIH) in the U.S.,which extracted anonymized patient/newborn data from the electronic medical records. As this study did not collect additional information from patients, the Ethics Committees at NIH and all collaborating centers waived the requirement for informed consent from the patients. The data are publicly available from NIH. A secondary data analysis using anonymized, publicly available data for research is exempted from the ethics review at the Xinhua Hospital, Shanghai Jiao Tong University School of Medicine.

## Results

Table 1 presents the baseline characteristics of patients and the intrapartum oxytocin regimens by parity. A high dose regimen was used in 58% of nulliparas and 57% of multiparas. 3% of nulliparas and 2% of multiparas achieved a highest dose >20 mU/min. The intrauterine pressure catheter was used in 38% and 20% women, respectively. The vast majority of women used epidural analgesia in both nulliparas and multiparas.

**Table 1:** Baseline characteristics of the study population by parity.

	Nulliparas	Multiparas
	(n = 4107)	(n = 4881)
BMI at admission to delivery (Mean±SD) <18.5 (%) 18.5–24.9 (%) 25–29.9 (%) 30–34.9(%) ≥35 (%)	29.3(4.9) 127(3%) 659(16%) 1836(45%) 1024(25%) 461(11%)	29.8(5.3) 131(3%) 779(16%) 2043(42%) 1216(25%) 712(15%)
Maternal age (Mean±SD)	24.1(4.4)	28.6(4.8)
Starting dose (mU/min)		
Low dose (1,2 or 3)	1713(42%)	2100(43%)
High dose (4)	2394(58%)	2781(57%)
Highest dose (mU/min.)		
1–10	2285(55%)	3069(63%)
11–20	1712(42%)	1704(35%)
>20	110(3%)	108(2%)
Use of intrauterine pressure catheter	1529(37%)	967(20%)
Epidural analgesia	3987(97%)	4555(93%)

Table 2 shows the duration of labor for cervical dilation to the next centimeter from 4 to 10 cm in nulliparas and multiparas when the oxytocin was started at that interval. The duration of cervical dilation from 4 to 5 cm, when oxytocin just started, could be very long, with a median of 3 hours and a 95^th^ percentile up to 10 hours. The duration became progressively shorter as labor advanced, particularly so in multiparas.

**Table 2:** Duration of labor for cervical dilation to the next centimeter with oxytocin starting at the beginning of the interval*.

Starting oxytocin at the Interval	nulliparas		multiparas	
	N	Duration (h) 50^th^ (95^th^) percentile	N	Duration (h) 50^th^ (95^th^) percentile
4–5 cm	740	2.9 (8.8)	850	3.1 (10.1)
5–6 cm	460	1.7 (5.8)	646	1.9 (8.0)
6–7 cm	319	1.4 (5.2)	445	1.3 (6.1)
7–8 cm	221	1.1 (5.0)	316	1.0 (4.6)
8–9 cm	139	1.5 (6.4)	219	0.9 (3.8)
9–10 cm	105	1.8 (5.5)	150	0.7 (2.9)
6–10 cm	319	2.1 (6.0)	445	1.7 (6.2)

*Interval censored regression.

Table 3 presents duration of labor for cervical dilation to the next centimeter in women whose oxytocin had reached the highest dose. From 4-5cm, it might take more than 2hours (95^th^ centiles) to reach to the next centimeter in nulliparas. After 5cm, labor accelerated much faster in all groups. In fact, all durations at the 95th centiles were less than 2 hours (95th centiles).

**Table 3:** Duration of labor for cervical dilation to the next centimeter with oxytocin reaching the highest dose before the start of the interval*.

Interval	Nulliparas		Multiparas	
	N	Duration (h), 50^th^ (95^th^) percentile	N	Duration (h), 50^th^ (95^th^) percentile
4–5 cm	967	0.7 (2.4)	551	0.6 (1.9)
5–6 cm	1596	0.5 (1.5)	1188	0.4 (1.1)
6–7 cm	2126	0.4 (1.0)	1848	0.3 (0.8)
7–8 cm	2533	0.4 (1.0)	2434	0.3 (0.6)
8–9 cm	2917	0.4 (0.9)	2958	0.2 (0.5)
9–10 cm	3200	0.5 (1.6)	3362	0.2 (0.6)
6–10 cm	2127	0.5 (1.5)	1855	0.4 (0.9)
2^nd^ stage without epidural analgesia	89	0.5 (2.2)	212	0.1 (0.4)
2^nd^ stage with epidural analgesia	3340	1.2 (3.1)	3452	0.4 (1.1)

* Interval censored regression.

We separated low- and high-dose oxytocin regimens and repeated the above analyses in Tables 2 and 3. Although women with a high-dose regimen for augmentation tended to have slightly faster labor than those with a low-dose regimen, the difference was not substantial (S1–S4 Tables).

## Discussion

Our analysis shows that when oxytocin is just started for labor augmentation in the early first stage, it may take a long time for the cervix to dilate by 1 cm. However, after 5 cm dilation and effective uterine contractions have been achieved, the vast majority of labors that were ended with vaginal delivery and normal perinatal outcomes took less than 2 hours to progress by 1 cm dilation or more.

Dystocia is a major cause for cesarean deliveries worldwide. For example, up to 34% of cesarean deliveries performed on nulliparas and 60% on multiparas in the US are due to dystocia or arrest of dilation in the first stage of labor.[1,8] When oxytocin is used after 6cm, current guidelines recommend allowing a minimum of 4 hours of effective uterine contractions or at least 6 hours of oxytocin augmentation with ineffective uterine activity and no cervical change before labor arrest is diagnosed.[3] Yet, little evidence is available concerning the optimal waiting time for augmentation under different cervical dilations before failure of augmentation is considered.

Prolonged labor is associated with postpartum hemorrhage, chorioamnionitis, cesarean delivery, birth trauma, NICU admission and other neonatal complications. A study of 5388 women found that prior to a cervical dilatation of 6 centimeters, women induced or augmented with oxytocin, may spend up to 10 hours to achieve each 1cm of dilation, compared to women whose labor was spontaneous.[9] A prior study by Chen et al suggested that in women who underwent induction of labor, the risk for maternal morbidity and cesarean delivery remained increased when the length of the first stage was longer than 24 hours compared to women with a first stage between 0–12 hours.[10]

Our large observational study demonstrates that in parturients treated with oxytocin for labor augmentation it may take many hours to achieve effective uterine contractions and lead to cervical change even after 3 cm dilation, which is consistent with prior studies. We also found that once the effective uterine contraction was achieved in late first stage, labor progressed quickly. For instance, after 5 cm dilation 95% of women had cervical dilation to the next centimeter in less than 2hours. These patterns were consistent with those in nulliparous and multiparous women of spontaneous labor and deliveries without oxytocin induction or augmentation.[11]

Rouse et al. conducted two prospective observational studies in 554 and 501 women with a diagnosis of labor arrest at ≥3 cm cervical dilation, respectively.[12,13] After 2 hours of oxytocin use with adequate uterine contraction (defined as a uterine contraction pattern of greater than 200 Montevideo units), 91% and 92% of the women had labor progression, respectively. By extending to 4 hours of oxytocin use with adequate uterine contraction, additional 5% of women had vaginal delivery. Considering that our study excluded all cesarean deliveries, our findings are consistent with those of Rouse et al.

However, our study differs from previous studies to some extent. Rouse et al. diagnosed dystocia at cervical dilation of at least 3 cm and complete effacement, or 4 cm and at least 75% effacement with uterine contractions at least two in 10 minutes, and oxytocin was then started.[13] It was not clear how long it took before achieving an effective uterine contraction and at what cervical dilation effective contractions were achieved. It would be interesting to know how many of those additional 5% of women who had vaginal delivery after 4 hours of oxytocin had their initial diagnosis of augmentation failure at 3 or 4 cm dilation, i.e., how many of these additional savings came from women of labor tardiness in early stage of labor. Our data support that at the pre-active labor the 4-hour rule is justified. But when the labor is more advanced (after cervical dilation beyond 5 or 6 cm), is 4 hours still required before the diagnosis of labor arrest is considered? A further analysis of the previous data may shed light on this issue. There may also be a trade-off between additional savings and the potential side effects of prolonged labor (e.g., maternal infection). [12,13] Finally, low- and high-dose oxytocin regimens appeared to have had similar effects in labor augmentation.

The effect of oxytocin for labor augmentation appears somehow blunted by obesity. One study including women with spontaneous onset of labor who had either cesarean or vaginal deliveries, found that oxytocin augmentation was less effective among obese compared to normal-weight women, more often failing to prevent unplanned cesarean delivery for slow labor progress.[14] Additionally, Carlson et al.[15] showed that even in term, spontaneously laboring, healthy, nulliparous women with no prelabor rupture of membranes, obese women with higher BMIs had significantly increased mean oxytocin infusion rates compared to obese women with lower BMIs. Further, increased maternal BMI independently predicted higher hourly oxytocin doses. Nonetheless, Kominiarek et al.[16] showed that substantial slow labor progression was seen mostly in women with a BMI ≥35 kg/m^2^.

Although the exact mechanism remains unclear, the hormonal milieu of obese women, which appears to interact with oxytocin regulation and response, may affect myometrium contractility, as well as the variation in expression and function of the oxytocin receptor of the human myometrium caused by increased BMI.[17] This may explain in part why obese women may have a longer labor. Unfortunately, we did not have a sufficient number of very obese women for a stratified analysis for labor progression by BMI. Our previous study showed that only when BMI was ≥35 or 40 kg/m^2^ did the labor progression differ substantially from that in normal weight women and 95% of women would have progressed for 1 cm after 2 hours that after 6 or 7 cm, even in very obese women.[16] More studies are needed to quantify the labor progression in obese women and determine whether obese women need a higher dose of oxytocin.

Our study has limitations. First, we focused on the duration of labor under oxytocin for augmentation in women with normal perinatal outcomes. We excluded births of abnormal outcomes to ensure that our observations were within acceptable safety. We also excluded cesarean deliveries because we did not have a standard labor management protocol for the study. We suspect that some of the cesarean deliveries may have been performed prematurely, which could affect the estimates of duration of labor. Second, we could not employ a uniform definition of “effective uterine contraction” in our study. Instead, we assumed that when the oxytocin dose reached the highest dose, the woman had achieved effective uterine contraction. This assumption and related uncertainty may have increased the variance of labor duration in our study. Finally, some women cannot achieve effective uterine contraction despite oxytocin stimulation due to oxytocin receptor variants. [18] Exposure to oxytocin doses greater than 20 mu/min is uncommon among women; they are more likely to have a cesarean delivery. [19] Thus, our conclusions may not be applicable to these women.

## Conclusion

Our analysis of data from a large, contemporary cohort of women managed with oxytocin in labor for dystocia has three important clinical messages. First, it may still be too soon to diagnose labor arrest after a 4–6 hour interval following initiation of oxytocin. More time is required to ensure that oxytocin achieves an optimal effect, especially during early first stage of labor. Second, once effective uterine contractions are achieved in women with cervical dilation of 5 cm or more, more than 95% of them progress to the next centimeter of dilation in less than 2 hours. And finally, high- and low-dose oxytocin had a similar impact on labor progression in augmented labor. Further prospective observational studies or randomized clinical trials are needed to confirm our findings.

## Supporting information

S1 Table Duration of labor for cervical dilation to the next centimeter with oxytocin starting at the interval (low starting dose). (DOCX)

S2 Table Duration of labor for cervical dilation to the next centimeter with oxytocin starting at the interval (high starting dose). (DOCX)

S3 Table Duration of labor for cervical dilation to the next centimeter with oxytocin reaching the highest dose before the start of the interval*(low starting dose). (DOCX)

S4 Table Duration of labor for cervical dilation to the next centimeter with oxytocin reaching the highest dose before the start of the interval*(high starting dose). (DOCX)
