# Supplementary material for: The expected labor progression after labor augmentation with oxytocin: A retrospective cohort study
Source: PLoS One. 2018 Oct 31;13(10):e0205735. doi: 10.1371/journal.pone.0205735 (PMC6209192; doi:10.1371/journal.pone.0205735)
Supplement: S1 Table — (DOCX) [file pone.0205735.s001.docx]

S1 Table. Duration of labor for cervical dilation to the next centimeter with oxytocin starting at the interval (low starting dose)

| Starting oxytocin at the Interval | nulliparas | | multiparas | |
| --- | --- | --- | --- | --- |
|  | N | Duration (h)  50^th^ (95^th^) percentile | N | Duration (h)  50^th^ (95^th^ ) percentile |
| 4 – 5 cm | 271 | 2.9 (7.9) | 335 | 3.4 (11.7) |
| 5 – 6 cm | 186 | 1.9 (5.8) | 301 | 2.0 (8.1) |
| 6 – 7 cm | 158 | 1.6 (5.5) | 197 | 1.4 (6.7) |
| 7 – 8 cm | 131 | 1.3 (5.7) | 155 | 1.0 (4.5) |
| 8 – 9 cm  9 – 10 cm | 74 | 1.7 (6.1) | 108 | 1.0 (4.1) |
|  | 63 | 1.9 (5.4) | 76 | 0.8 (3.1) |
| 6 – 10 cm | 158 | 2.2 (6.3) | 197 | 1.8 (7.1) |
