# Supplementary material for: The expected labor progression after labor augmentation with oxytocin: A retrospective cohort study
Source: PLoS One. 2018 Oct 31;13(10):e0205735. doi: 10.1371/journal.pone.0205735 (PMC6209192; doi:10.1371/journal.pone.0205735)
Supplement: S2 Table — (DOCX) [file pone.0205735.s002.docx]

S2 Table. Duration of labor for cervical dilation to the next centimeter with oxytocin starting at the interval (high starting dose)

| Starting oxytocin at the Interval | nulliparas | | multiparas | |
| --- | --- | --- | --- | --- |
|  | N | Duration (h)  50^th^ (95^th^) percentile | N | Duration (h)  50^th^ (95^th^ ) percentile |
| 4 – 5 cm | 469 | 3.0 (9.4) | 515 | 2.8 (8.7) |
| 5 – 6 cm | 274 | 1.6 (5.7) | 345 | 1.9 (7.8) |
| 6 – 7 cm | 161 | 1.3 (4.9) | 248 | 1.3 (5.7) |
| 7 – 8 cm | 90 | 0.9 (3.7) | 161 | 1.0 (4.7) |
| 8 – 9 cm  9 – 10 cm | 65 | 1.3 (6.2) | 111 | 0.8 (3.6) |
|  | 42 | 1.7 (5.6) | 74 | 0.7 (2.7) |
| 6 – 10 cm | 161 | 2.0 (5.6) | 248 | 1.6 (5.4) |
