# Supplementary material for: The expected labor progression after labor augmentation with oxytocin: A retrospective cohort study
Source: PLoS One. 2018 Oct 31;13(10):e0205735. doi: 10.1371/journal.pone.0205735 (PMC6209192; doi:10.1371/journal.pone.0205735)
Supplement: S3 Table — (DOCX) [file pone.0205735.s003.docx]

S3 Table. Duration of labor for cervical dilation to the next centimeter with oxytocin reaching the highest dose before the start of the interval^*^(low starting dose)

| Interval | Nulliparas | | Multiparas | |
| --- | --- | --- | --- | --- |
|  | N | Duration (h), 50^th^ (95^th^) percentile | N | Duration (h), 50^th^ (95^th^) percentile |
| 4 – 5 cm | 302 | 0.7 (2.6) | 192 | 0.5 (1.3) |
| 5 – 6 cm | 522 | 0.5 (1.3) | 409 | 0.4 (1.1) |
| 6 – 7 cm | 731 | 0.4 (0.9) | 685 | 0.3 (0.7) |
| 7 – 8 cm | 905 | 0.4 (1.0) | 960 | 0.3 (0.6) |
| 8 – 9 cm | 1087 | 0.4 (1.0) | 1212 | 0.2 (0.5) |
| 9 – 10 cm | 1238 | 0.5 (1.6) | 1403 | 0.3 (0.6) |
| 6 – 10 cm | 731 | 0.5 (1.4) | 688 | 0.3 (0.9) |
| 2^nd^ stage without epidural analgesia | 53 | 0.5 (2.5) | 104 | 0.1 (0.4) |
| 2^nd^ stage with epidural analgesia | 1310 | 1.2 (3.1) | 1469 | 0.4 (1.2) |

^*^ Interval censored regression.
