# Supplementary material for: The expected labor progression after labor augmentation with oxytocin: A retrospective cohort study
Source: PLoS One. 2018 Oct 31;13(10):e0205735. doi: 10.1371/journal.pone.0205735 (PMC6209192; doi:10.1371/journal.pone.0205735)
Supplement: S4 Table — (DOCX) [file pone.0205735.s004.docx]

S4 Table . Duration of labor for cervical dilation to the next centimeter with oxytocin reaching the highest dose before the start of the interval^*^(high starting dose)

| Interval | Nulliparas | | Multiparas | |
| --- | --- | --- | --- | --- |
|  | N | Duration (h), 50^th^ (95^th^) percentile | N | Duration (h), 50^th^ (95^th^) percentile |
| 4 – 5 cm | 665 | 0.7 (2.3) | 359 | 0.6 (2.2) |
| 5 – 6 cm | 1074 | 0.5 (1.5) | 779 | 0.4 (1.1) |
| 6 – 7 cm | 1395 | 0.4 (1.1) | 1163 | 0.3 (0.8) |
| 7 – 8 cm | 1628 | 0.4 (0.9) | 1474 | 0.2 (0.6) |
| 8 – 9 cm | 1830 | 0.3 (0.9) | 1746 | 0.2 (0.4) |
| 9 – 10 cm | 1962 | 0.5 (1.7) | 1959 | 0.2 (0.6) |
| 6 – 10 cm | 1396 | 0.6 (1.6) | 1167 | 0.4 (0.9) |
| 2^nd^ stage without epidural analgesia | 36 | 0.4 (2.2) | 108 | 0.1 (0.4) |
| 2^nd^ stage with epidural analgesia | 2030 | 1.1 (3.0) | 1983 | 0.4 (1.1) |

^*^ Interval censored regression.
